# Perspectives on optimizing care of patients in multidisciplinary chronic kidney disease clinics

**DOI:** 10.1186/s40697-016-0122-9

**Published:** 2016-05-12

**Authors:** David Collister, Randall Russell, Josee Verdon, Monica Beaulieu, Adeera Levin

**Affiliations:** Section of Nephrology, University of Manitoba, Winnipeg, MB Canada; The Ottawa Hospital, Ottawa, ON Canada; Division of Geriatric Medicine, McGill University, Montreal, QC Canada; Division of Nephrology, University of British Columbia, Vancouver, BC Canada

**Keywords:** Multidisciplinary, Chronic kidney disease, Clinics, Communication, Standardized operating procedures, Scorecards

## Abstract

**Purpose of review:**

To summarize a jointly held symposium by the Canadian Society of Nephrology (CSN), the Canadian Association of Nephrology Administrators (CANA), and the Canadian Kidney Knowledge Translation and Generation Network (CANN-NET) entitled “Perspectives on Optimizing Care of Patients in Multidisciplinary Chronic Kidney Disease (CKD) Clinics” that was held on April 24, 2015, in Montreal, Quebec.

**Sources of information:**

The panel consisted of a variety of members from across Canada including a multidisciplinary CKD clinic patient (Randall Russell), nephrology fellow (Dr. David Collister), geriatrician (Dr. Josee Verdon), and nephrologists (Dr. Monica Beaulieu, Dr. Adeera Levin).

**Findings:**

The objectives of the symposium were (1) to gain an understanding of the goals of care for CKD patients, (2) to gain an appreciation of different perspectives regarding optimal care for patients with CKD, (3) to examine the components required for optimal care including education strategies, structures, and tools, and (4) to describe a framework and metrics for CKD care which respect patient and system needs. This article summarizes the key concepts discussed at the symposium from a patient and physician perspectives. Key messages include (1) understanding patient values and preferences is important as it provides a framework as to what to prioritize in multidisciplinary CKD clinic and provincial renal program models, (2) barriers to effective communication and education are common in the elderly, and adaptive strategies to limit their influence are critical to improve adherence and facilitate shared decision-making, (3) the use of standardized operating procedures (SOPs) improves efficiency and minimizes practice variability among health care practitioners, and (4) CKD scorecards with standardized system processes are useful in approaching variability as well as measuring and improving patient outcomes.

**Limitations:**

The perspectives provided may not be applicable across centers given the differences in patient populations including age, ethnicity, culture, language, socioeconomic status, education, and multidisciplinary CKD clinic structure and function.

**Implications:**

Knowledge transmission by collaborative interprovincial and interprofessional networks may play a role in facilitating optimal CKD care. Validation of system and clinic models that improve outcomes is needed prior to disseminating these best practices.

## Abrégé

**Objectif de la revue:**

Cette revue se veut une récapitulation des thèmes abordés lors du colloque intitulé *« Perspectives on Optimizing Care of Patients in Multidisciplinary Chronic Kidney Disease Clinics »*. Ce colloque organisé conjointement par la Société Canadienne de Néphrologie, la *Canadian Association of Nephrology Administrators* (CANA) et la *Canadian Kidney Knowledge Translation and Generation Network* (CANN-NET), s’est tenu le 24 avril 2015 à Montréal, au Canada.

**Sources:**

Cette table ronde réunissait des membres provenant de partout au Canada. Les intervenants invités à discuter lors de ce colloque comptaient un patient fréquentant une clinique multidisciplinaire en suivi des maladies rénales chroniques (Randall Russell) un chercheur boursier en néphrologie (Dr David Collister), une gériatre (Dre Josée Verdon) et deux néphrologues (Dre Monica Beaulieu et Dre Adeera Levin).

**Observations:**

Ce colloque visait plusieurs objectifs. D’abord on voulait se faire une meilleure idée des objectifs fixés en matière de soins offerts aux patients souffrant d’IRC. On a ensuite tenté de faire le portrait des différents points de vue en matière de soins optimaux à prodiguer aux patients atteints d’IRC et se pencher sur les éléments requis pour y arriver, notamment les structures et outils nécessaires, et les méthodes pédagogiques à favoriser. Finalement, ce colloque visait à définir un cadre et des paramètres de soins en IRC qui respectent les besoins des patients et du système de santé. Le présent article résume les concepts-clés discutés lors de ce colloque du point de vue du médecin traitant, mais également de celui d’un patient atteint d’IRC.

Les messages-clés abordés incluent les observations suivantes:

1) Il est important de tenir compte des valeurs et des préférences du patient dans l’établissement des priorités des cliniques multidisciplinaires et des modèles de programmes provinciaux en IRC.

2) On constate que les obstacles à une communication et à un enseignement efficaces sont fréquents chez les patients âgés. Ainsi les stratégies adaptatives limitant leur influence sont cruciales pour améliorer l’adhésion du patient au traitement et faciliter la prise de décision conjointe.

3) On observe que l’usage de procédures opérationnelles normalisées améliore l’efficacité et minimise la variabilité dans la pratique chez les professionnels de la santé.

4) Les fiches d’évaluation en IRC doublées d’une uniformisation des systèmes et des procédés, sont utiles pour aborder le traitement de la variabilité tout autant que pour mesurer et améliorer les résultats pour les patients.

**Limites de l’étude:**

Les points de vue exprimés peuvent ne pas s’appliquer dans tous les centres de soins compte tenu des différences appréciables parmi les patients souffrant d’IRC. Ces différences incluent notamment l’âge l’origine ethnique, les différences culturelles, la langue parlée, le statut socio-économique, le niveau de scolarité, de même que la structure et les fonctions de la clinique multidisciplinaire de suivi en IRC fréquentée par le patient.

**Conclusion:**

La transmission des connaissances par l’entremise d’un réseau interprovincial et interprofessionnel de collaboration pourrait contribuer à faciliter l’administration de soins optimaux en IRC. Une validation du système et des modèles cliniques permettant l’amélioration des résultats pour les patients est requise préalablement à la diffusion de ces pratiques exemplaires.

## What was known before

Multidisciplinary CKD clinics improve patient outcomes, but there is variability in clinic structure and function across Canada. Exploring optimal CKD patient care practices from the patient, physician, and provincial renal program perspective is important in the development of multidisciplinary CKD clinics and to identify what practices are effective in improving outcomes.

## What this adds

Incorporating patient values and preferences, employing effective communication and education strategies, adopting SOPs, and utilizing CKD scorecards are all practices that are valuable in improving the care of patients in multidisciplinary CKD clinic settings.

## Background

CKD is a global public health concern that is increasing in incidence and prevalence. It is estimated that 15 % of Canadians have CKD [1], and this epidemic is driven by the elderly with significant comorbidities [2]. There is a degree of variability in disease burden across Canada. The care of the CKD population is complex and requires many interactions between the patient, family, primary care provider, and multidisciplinary CKD clinic team as well as several inpatient and outpatient services. Optimal care is generally defined as care that leads to the best outcomes for the individual, population, and society; it is the goal of any health care system. However, patient-centered outcomes such as engagement, symptom control, and satisfaction may not necessarily align with the physician-centric priorities of slowing the progression of CKD, achieving clinical targets, and improving morbidity and mortality [3, 4]. Regardless, clinicians strive to deliver effective and efficient care with the goals of identifying, risk stratifying, educating, and managing patients with CKD with appropriate preparation and transition to end-stage renal disease (ESRD) with renal replacement therapy (RRT: dialysis or transplantation) or conservative therapy. The concept of shared decision-making [5] has gained acceptance in most clinical jurisdictions in this regard.

## Review

### Patient values and perspectives

Understanding patient values and exploring their perspectives are critical to caring for the CKD population [6–8]. Randall Russell provided a contextual framework by sharing his personal journey as a CKD patient transitioning from his primary nephrologist in the community to the Progressive Renal Insufficiency Clinic at The Ottawa Hospital. Initially, he felt anxiety regarding his illness trajectory and the transition between clinic models but ultimately viewed the experience as motivating and empowering. His priorities as a CKD patient include continuity through longitudinal care, accessibility, and the sense of support from all members of the multidisciplinary team. He values autonomy in decision-making [9] and acquiring knowledge [10] through renal education with clear and comprehensive information. The availability of the multidisciplinary team members outside of clinic appointments is also important to him. Lastly, he shared his gratefulness for healthcare engagement in improving CKD care [11] and encouraged the active participation of all CKD patients in their care. However, he may not be representative of the entire Canadian CKD population given its diversity in age, ethnicity, culture, language, socioeconomic status and education. Tong et al [12] identified 5 themes in CKD patient preferences and experiences including personal meaning of CKD, managing and monitoring health, lifestyle consequences, family impact and informal support structures. 5 other themes emerged in adolescents and young adults [13] including inferiority, insecurity, injustice, resilience and adjustment mentality. In the elderly [14], there is shock about a diagnosis, uncertainty about disease progression and a lack of preparation for living with dialysis. Thus, individualizing care by exploring the patient’s values and perspectives is important in improving their well-being and satisfaction. 

### Principles of care models for older adults

The principles of care models designed for the elderly have relevance to the CKD population given that a significant portion of this population is considered elderly from an aging or biologic perspective [2, 15, 16]. Normal aging affects senses (vision, hearing, touch, reaction) and functions (cognition, spatial orientation, motor coordination, mobility, work rate, working memory, executive function, motor coordination and mobility) [17], which may create barriers to communication and education. Screening for sensory deficits [18, 19], intervening with hearing or visual aids, and using other techniques (adequate lighting, appropriate sized print, adequate voice intensity, multimodal cues) may attenuate these barriers. Mood disorders [20, 21] and cognitive impairment are common in CKD [22, 23] patients and the elderly. Thus, formally screening for anxiety, depression [24] and cognitive impairment [25] on a routine basis (or alternatively if a threshold pre-test probability exists) may be valuable, as these conditions may negatively impact patient interaction and ability to retain information presented. Compliance can be improved by simplifying instructions, reinforcing behavior on a regular basis and by checking/rechecking comprehension. Involving a caregiver in all clinic visits is also crucial to corroborate illness trajectory and may improve adherence. As cognitive functions such attention, concentration, comprehension and retention may be impaired, strategies to enhance communication are frequently necessary. These may include the use of direct, concrete and actional language as well as “right branching” sentences (see Table 1). Information should be broken down into simple elements with each explained separately using techniques to ensure attention and retention of information such as “teach-back”, utilizing multiple senses (e.g. oral and written instructions), and the repetition of concepts over many sessions [26, 27]. Ideally, education sessions should last less than 15 minutes and only address 3-5 points at a time to maximize concentration and retention. Renal education should also be individually tailored in format, length, frequency, and size (group vs. individual) using a patient-centered approach addressing feasibility and acceptability. Lastly, deficits in health literacy are common in the CKD population [28] so clinicians must be sensitive with their use of language complexity and terminology in all forms of communication [29]. Given the diversity of the CKD population across Canada, a tailored approach to these principles of care are needed to promote health literacy, learning and understanding, As Canada is a multilingual country, translators should be available during clinic visits and if not, caregivers can be utilized instead if language barriers exist. Additionally, educational materials including pamphlets, posters and education sessions should be offered in the languages most prevalent in the population.

**Table 1:** Principles of care for older adults

Barrier	Identification	Strategies
Sensory deficits	Screening for visual acuity and hearing loss formally, informally	-Referral for aids (glasses, hearing aids)
		-Optimize the learning environment (adequate lighting, minimize glare, limit background noise)
		-Written instructions with large font sizing and multimodal information (visual and verbal through writing, pictogram, hands-on experience, videos, web-links, online)
		-Appropriate voice intensity, pitch, pacing, eye level, direct visualization to allow for lip reading
Cognitive impairment	Screening with MMSE, MoCA, clock drawing, cognitive battery testing	-Breakdown information into small units (focus on only 3–5 issues or ess per session, <15 minutes per session)
		-Explain each element separately
		-Direct, actional, concrete language (“take one tablet in the morning and one at night” not “take twice a day”)
		-Individualized, tailored educational sessions
		-“Right branching” (“take a seat and you won’t miss the session” not “if you don’t want to miss the session, take a seat”)
		-Teach-back technique
		-Involvement of caregiver
		-Refer for treatment as indicated
Mood disorders	Screening formally, informally	-Reassurance
		-Simplify
		-Pacing
		-Refer for treatment as indicated (medications, CBT)
Health literacy	Assuming baseline limited health literacy vs. screening	-Limiting language complexity
		-The use of appropriate terminology in all forms and venues of communications (“high blood pressure” not “hypertension”)
Adherence	“How many times have you missed (behavior) in the last week?”	-Simplify
		-Explain (indications, consequences, prioritization)
		-Reinforce
		-Checking/rechecking understanding
		-Address feasibility, acceptability
		-Involvement of caregiver

*MMSE* Mini Mental Status Examination, *MoCA* Montreal cognitive assessment, *CBT* cognitive behavioral therapy

### Standardized operating procedures for physicians and multidisciplinary team members: defining inputs and outputs

Multidisciplinary CKD clinics improve clinical targets (blood pressure, ACE/ARB use, hemogloblin, calcium, phosphate, bicarbonate) and outcomes (rate of eGFR decline, acute RRT, vascular access, hospitalizations, mortality, costs) in both adult [30–36] and pediatric populations [37, 38]. However, it remains uncertain how to optimally structure multidisciplinary CKD clinics and what resources should be allocated to promote their operation. CKD care is highly variable across Canada by referral, entry, staffing, resources, focus, size, and efficiency [39]. This context in which the care of CKD patients is delivered influences quality but differs from province to province and center to center depending on individual program scope and current practices.  Process improvement is defined as a series of action taken to identify, analyze, and improve existing processes within an organization to meet goals and objectives [40]. Process engineering (the identification of inputs, operations and outputs for any process) for a multidisciplinary CKD clinic involves clerks, nurses, dietician, pharmacists, physicians, rooms, equipment and actions required to ensure healthy and satisfied CKD patients. In a multidisciplinary stage 4/5 CKD clinic in Winnipeg, Manitoba [41], there was a redundancy in tasks and poor communication among the team with significant “down time” and wait times for patients and no clear dynamic monitoring of clinical and administrative outcomes. A time study and task consistency analysis demonstrated heterogeneity in practice. A sequence of patient flow through the clinic was established with 15 minutes allocated per encounter, SOPs for all multidisciplinary team members were created focusing on core competencies after focus group discussions and a new clinic record was created based on these SOPs. The goal of the clinic redesign by process engineering was to eliminate bottlenecks, improve patient flow and standardize quality of care through the elimination occupational uncertainty. A pre/post time study, task analysis and chart review for quality of patient care parameters was performed. Mean throughput times (time for a patient to progress through the clinic) decreased and the standard deviation of mean cycle times and physician cycle time decreased with adherence to time standards. There was less variability of task performance and no changes in clinical targets but there was an association with favorable outcomes. SOPs play an important role in multidisciplinary CKD clinics to optimize quality, efficiency and accountability.

### Framework and goals of care: CKD scorecards

The BC Renal Agency Provincial Kidney Care Committee’s (KCC) goal is to provide infrastructure and mechanisms to facilitate a provincial and interprofessional approach to improvements in CKD care [42]. Since the establishment of the provincial KCC in 2011, the group has involved all provincial health authorities in the creation of a formal framework including definitions, best practice documents, and a set of metrics to ensure accountability and enable quality improvement. There is a systematic gathering of data using a provincial database, which permits a description of provincial CKD clinic demographics, comorbidities, and achievement of clinical targets and outcomes. In collaboration with provincial health authorities, KCC developed a work plan that included the creation of a document entitled “Best Practices in Organizing Kidney Care” (www.bcrenalagency.ca) that outlines guidelines, protocols, and algorithms for ordering and reviewing of bloodwork, medication reconciliation, and modality education. The group has also defined the goals of CKD clinics, referral and repatriation criteria, and interprofessional team members’ roles and responsibilities. In addition, the pathways for transitions between CKD and RRT modalities (hemodialysis, peritoneal dialysis, and renal transplantation) are well articulated, defining the roles for various team members.

A scorecard approach in health care terms refers to the process of formally adjudicating systems for benchmarks of quality of care defined by guidelines. Its strengths include standardized and mandatory reporting with comparisons across centers with the potential for goal setting and improvement in outcomes. The KCC has developed and reported CKD scorecards for all clinics in an unblinded manner after establishing a set of indicators of quality of care and goals linked to best practices. For example, hemoglobin and iron target achievements would reflect implementation of anemia protocol; ACE/ARB use would reflect recommended best practice for delay of renal progression and cardiovascular health; the proportion of patients with eGFR<20ml/min and documented planned modality would indicate appropriate timing of education; the proportion of patients starting on the modality of their choice indicate appropriate timing and preparation; and independent modality rates of those attending clinics would be an ‘integrated’ measure of the entire process of care, including appropriate access creation and education, decision making and system functioning. Each of these measures can be mapped to a specific set of activities important to patient outcomes and system functioning. The value of the KCC provincial approach is that it has permitted knowledge translation, transparency, and standardization of CKD care with the use of the “plan, study, do, act” cycle as an iterative process. Future goals are to include measures of patient oriented outcomes and other relevant metrics, and incorporate the assessment of how to address depression/anxiety, end of life, and advanced care planning activities into future metrics. Unfortunately, a limitation of scorecards is the need for the infrastructure for information management. This is currently available through provincially based CKD information systems in some regions but may not be readily available so alternatives with their associated costs are needed to properly evaluate processes and outcomes.

## Conclusions

**Fig. 1:**
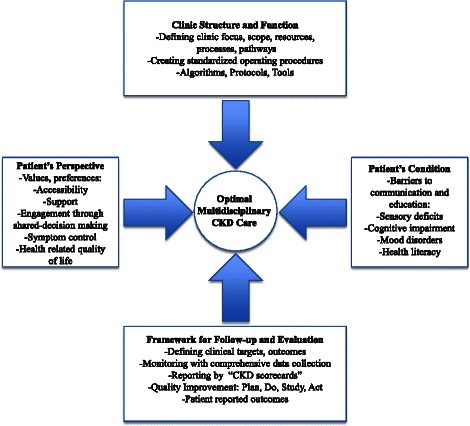
A framework for optimal multidisciplinary CKD care. *CKD* chronic kidney disease

The symposium presented perspectives from a patient, a geriatric physician, nephrology trainee, and nephrologists experiencing CKD and working within different provincial jurisdictions. Different perspectives in health care provision are important in understanding the current state and may lead to improvement through collaboration. Key learnings included the importance of incorporating patient values and preferences into planning multidisciplinary CKD clinic structure and function, the importance of deliberate use of strategies for effective communication and education in the elderly or those with impediments to learning (cognitive, psychological, physiological), and the value of adopting SOPs among team members and standardizing renal program processes to improve efficiencies. Within a provincial framework and with a robust information system, it is possible to monitor outcomes of both patients and the system using “CKD scorecards” as part of a continuous quality improvement cycle. The concepts and strategies described in the symposium are synergistic (see Fig. 1) and, if integrated into current existing systems, may serve as a template to improve the care of patients with CKD across Canada. Understanding the barriers and opportunities to implementation of standardized kidney care in different jurisdictions across Canada is an important future work.

## Abbreviations

ACE/ARB: angiotensin converting enzyme/angiotensin receptor blocker
CANA: Canadian Association of Nephrology Administrators
CKD: chronic kidney disease
CSN: Canadian Society of Nephrology
eGFR: estimated glomerular filtration rate
ESRD: end-stage renal disease
RRT: renal replacement therapy
SOPs: standardized operating procedures
